# Nonequivalence of Classical MHC Class I Loci in Ability to Direct Effective Antiviral Immunity

**DOI:** 10.1371/journal.ppat.1002541

**Published:** 2012-02-23

**Authors:** Kevin D. Pavelko, Yanice Mendez-Fernandez, Michael P. Bell, Michael J. Hansen, Aaron J. Johnson, Chella S. David, Moses Rodriguez, Larry R. Pease

**Affiliations:** 1 Department of Immunology, Mayo Clinic, Rochester, Minnesota, United States of America; 2 Department of Neurology, Mayo Clinic, Rochester, Minnesota, United States of America; Fox Chase Cancer Center, United States of America

## Abstract

Structural diversity in the peptide binding sites of the redundant classical MHC antigen presenting molecules is strongly selected in humans and mice. Although the encoded antigen presenting molecules overlap in antigen presenting function, differences in polymorphism at the MHC I *A*, *B* and *C* loci in humans and higher primates indicate these loci are not functionally equivalent. The structural basis of these differences is not known. We hypothesize that classical class I loci differ in their ability to direct effective immunity against intracellular pathogens. Using a picornavirus infection model and chimeric H-2 transgenes, we examined locus specific functional determinants distinguishing the ability of class I sister genes to direct effective anti viral immunity. Whereas, parental FVB and transgenic FVB mice expressing the *H-2K^b^* gene are highly susceptible to persisting Theiler's virus infection within the CNS and subsequent demyelination, mice expressing the *D^b^* transgene clear the virus and are protected from demyelination. Remarkably, animals expressing a chimeric transgene, comprised primarily of *K^b^* but encoding the peptide binding domain of D^b^, develop a robust anti viral CTL response yet fail to clear virus and develop significant demyelination. Differences in expression of the chimeric K^b^α1α2D^b^ gene (low) and D^b^ (high) in the CNS of infected mice mirror expression levels of their endogenous H-2^q^ counterparts in FVB mice. These findings demonstrate that locus specific elements other than those specifying peptide binding and T cell receptor interaction can determine ability to clear virus infection. This finding provides a basis for understanding locus-specific differences in MHC polymorphism, characterized best in human populations.

## Author Summary

MHC I genes are best understood as regulators of antiviral immunity. In humans and mice there are 2 to 3 homologous MHC I genes encoding highly polymorphic antigen presenting molecules which present virus proteins to T lymphocytes. A world wide effort has catalogued more than 6,300 classical *HLA* MHC I alleles in human populations, making these MHC loci among the best characterized polymorphic gene families. However, there has been little progress in understanding implications of the differences in polymorphism present at the *HLA A*, *B*, and *C* loci. By expressing MHC I molecules capable of presenting viral antigens under regulatory determinants from different sister MHC I genes of the mouse, we address the hypothesis that locus-specific differences in the regulation of the homologous MHC I sister genes can determine whether alleles at any particular locus can effectively target protective immunity against virus infection. We find that while the ability to activate cellular immune effectors is determined by the highly polymorphic MHC I sequences encoding the peptide binding domain, the ability of these T lymphocytes to effectively clear virus from the central nervous system can also be determined by gene sequences mapping outside of this region.

## Introduction

MHC class I antigen presenting molecules sample peptides generated intracellularly and present them on the surface of cells to CD8+ T cells bearing class I restricted T cell receptors [1]–[3]. The MHC class I multigene families in mice and humans contain three genes encoding classical antigen presenting molecules [4], generally considered to have redundant functions. These classical MHC I molecules direct immune responses, determine host resistance to disease, and are considered key variables in vaccine design. However, certain lines of evidence indicate that the classical HLA (*A*, *B*, and *C*) and H-2 (*K*, *D*, and *L*) genes may not strictly be redundant, but instead have distinctive functions. Delineating these differences would be important for understanding the roles of class I genes in disease and shape rational development of vaccines to prevent or treat viral infections and for the immunotherapy of cancers.

Immunogenetic data provide the primary support for the hypothesis that MHC class I classical genes do not function equivalently. MHC polymorphism is understood in the context of naturally selected variation in the ability of the immune system to deal with constantly changing challenges by pathogens. Allelic comparisons at the nucleotide level provide convincing evidence that natural selection for amino acid diversity in residues positioned to interact directly with bound peptide favors amino acid replacements over synonymous substitutions [5], [6]. The nature of MHC polymorphism in mammal population is best understood from meta analysis of world wide studies of human populations. The numbers of identified alleles in human and chimpanzee populations assigned to the *A*, *B*, and *C* loci [5]–[8] differ substantially (e.g. the 1641 *HLA B* alleles >1176 *A* alleles >808 *C* alleles as enumerated in the IMGT/HLA Database, European Bioinformatics Institute). The probability that any of these three loci has an equivalent number of alleles is less than 10^−15^. This difference in the numbers of alleles is a strong indication that the classical class I loci are not functioning equivalently. MHC polymorphism in South American Indian populations [9]–[14] provides a snapshot of the selective forces operating over the 15,000 years following the settling of the new world by their Asian ancestors [15]. In these Amerindian populations in which only 8 of the defined 36 *B* locus allelic superfamilies from the old world have been identified, 21 subfamily members have emerged (discounting identified old-world superfamily allelic prototypes such as B15:01 and B35:01). In contrast, 8 subfamily alleles at the *A* locus are present, representing 4 of the 21 old world allelic families, and just 1 new variant at the C locus. Again, this pattern strongly diverges from the null hypothesis that the numbers of *HLA A*, *B*, and *C* alleles are evolving at the same rate in South America (P = 0.0121 for comparison of *A* and *B*, and 0.0195 for comparison of *A* and *C*). A similar pattern of natural selection appears to be functioning in chimpanzee populations where the numbers and structural diversity among *Patr B* alleles is larger than found at the Patr *A* and C loci [7], [8]. The different selection pressures operating on the A, B, and C loci, noted as well by others [5], [16], provides a compelling argument that each of these immune response regulatory genes do not function equivalently.

Interpretations of the unequal numbers of alleles that have emerged at the class I loci in humans and chimpanzees have focused on their peptide binding properties, as it is well known that amino acid changes that affect peptide binding by MHC I molecules influence immune function. This argument focuses on functional differences distinguishing alleles of a single locus. Because there are more than 800 to 1,600 allelic variants at the three human loci with widely disparate abilities to bind and present peptides, explanations predicated on allele specific peptide binding properties seem unsatisfactory. Here, we propose a different explanation: *locus specific differences in gene expression determine the relative importance of the class I genes for survival, driving locus specific frequencies of emerging allelic variants for the classical MHC genes*. The implication is that the classical class I genes are not equally effective in directing immunity against certain pathogens, and therefore, may not be equally effective in targeting vaccine antigens against viruses and perhaps cancers. We propose that the *C* locus alleles will be less effective antigen presenting molecules than the *A* locus alleles, which in turn will be less effective than the *B* locus alleles. This hierarchy is noteworthy as most vaccines are designed by convenience to target the *A* locus antigen presenting molecules because of their more limited polymorphisms.

Because the antigen presenting function of MHC I molecules are determined by their highly polymorphic peptide binding domains, variation within allelic series will overshadow differences among the proteins derived from different loci. In the laboratory mouse, the structures of genes expressed in vivo can be readily manipulated, and we have used this property to illustrate the principle underlying our hypothesis.

In the mouse where large numbers of *H-2 K* and *D* classical class I alleles have been described, the genetic ability to control picornavirus-induced demyelinating disease in the spinal cord maps to the *D* locus class I gene cluster (*H-2 D* and *L*) and does not seem to be influenced by the myriad of alleles present at the *K* locus [14]. This suggests that ability to resist persistent infection by Theiler's murine encephalomyelitis virus (TMEV) might be an example of classical class I loci differing in ability to provide effective immunity against a viral pathogen. An extensive analysis of MHC mediated resistance to TMEV demonstrated that certain alleles of the *D* gene can direct virus-specific CD8+ T cell immunity that is responsible for clearing virus infection from the CNS [9], [10], [12]–[14]. Whereas only certain D region alleles provide protection against persisting virus infection, alleles of the sister locus *H-2K* never seem to matter. Our studies of gene conversion mediated interchange of coding sequences among the α1 and α2 coding sequences of MHC genes in the mouse indicate that natural history of these sister genes has resulted in a complete scrambling of sequence diversity [11], [17]–[19]. This implies that structural diversity in the peptide and T cell receptor interaction domains of the D and K molecules is not likely the source of their differential ability to direct an effective and protective immune response against TMEV infection. In humans where interlocus exchange is more limited [8], this situation is approximated by the large numbers of allelic variants at the *HLA A*, *B* and *C* loci encoding distinctive peptide binding sites.

There are two competing hypotheses explaining the immune response phenotypes of mouse strains susceptible and resistant to persisting TMEV infection. The first is that certain MHC I alleles are capable of effectively presenting viral peptides to the CD8+ T cell compartment, and these effective alleles happen by chance to belong to the series of class I genes encoded within *H-2D*. The second possibility is that *H-2D* and *H-2K* genes are functionally distinct, such that while some alleles of H-2D can direct effective virus immunity, alleles of H-2K are ineffective as a group, irrespective of their ability to bind viral peptides and present them to CD8+ T cells.

Here we show that structural attributes of *K* and *D* genes other than their coding sequences specifying peptide binding properties are responsible for their differential ability to direct protective immunity against a picornavirus infection. These differences influence the relative expression of *H-2K* and *D*. This finding provides structural evidence differentiating the functional properties of the classical class I loci and relates these differences to the ability to fight a virus infection. This framework provides a basis for understanding the diverging evolutionary histories of members of the “classical” MHC class I gene family in animal populations, and provides context to our understanding of the polymorphism differences evident at the human *HLA A*, *B*, and *C* loci.

## Results

### Viral clearance is determined by regions outside of the peptide binding domain of the H-2D^b^ gene

**Figure 1:**
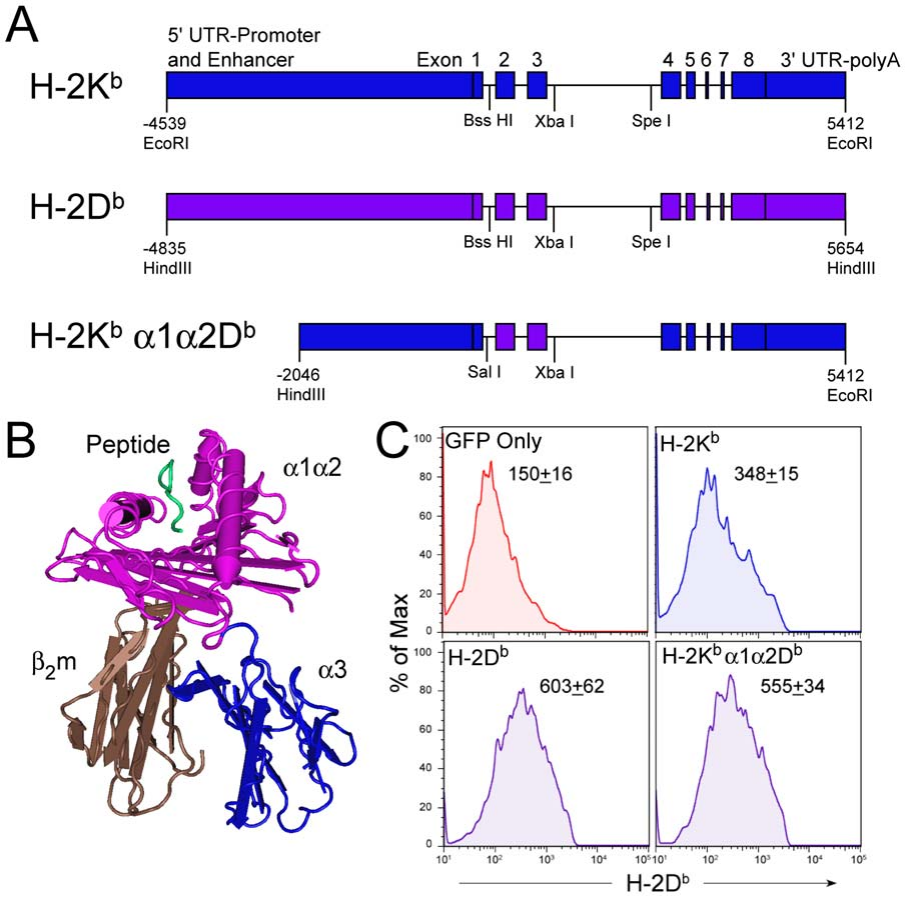
Generation of FVB K^b^α1α2D^b^ transgenic mice. (A) Segments of H-2K^b^ EcoRI and H-2D^b^ HindIII were used to generate a chimeric genomic construct with an H-2D^b^ Sal I/XbaI fragment on an H-2K^b^ backbone. (B) Expression of the construct yields a chimeric MHC class I molecule composed of the α1α2 domain from H-2D^b^ and the α3 domain from H-2K^b^. (C) Verification of H-2D^b^ and K^b^α1α2D^b^ transgene expression in 293T cells by flow cytometry. Data are mean fluorescence intensity of phycoerythrin labeled cells.

**Figure 2:**
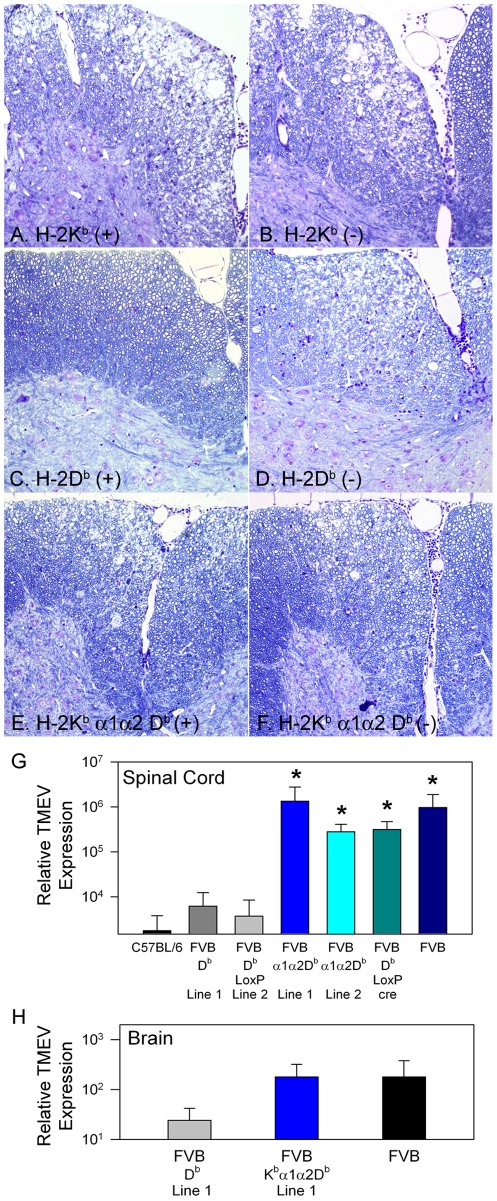
Spinal cord demyelination and persistent virus infection in FVB K^b^α1α2D^b^ transgenic mice. (A) Transgenic expression of H-2K^b^ fails to protect from TMEV induced demyelination, similar to non-transgenic control (B). (C) Expression of H-2D^b^ transgene protects FVB mice from TMEV induced demyelination present in littermate controls (D). (E) Expression of a chimeric K^b^α1α2D^b^ molecule fails to protect from demyelination, similar to non-transgenic (F). (G) Relative TMEV RNA levels in spinal cords from transgenic mice infected for 21 days (* p<0.05 by ANOVA). (H) Relative TMEV RNA levels in the brains of transgenic mice after 24 days of infection.

In order to study the structural properties of the H-2D and H-2K class I genes that are responsible for directing effective viral immunity against TMEV infection, we introduced genomic clones encoding the *K^b^* or *D^b^* (Figure 1A) genes into susceptible FVB mice. To control for possible gene integration positional effects, multiple independent founder lines were analyzed. One founder line of the *K^b^* transgene and three founder lines of the *D^b^* transgene were evaluated for susceptibility to TMEV induced demyelination. TMEV infected *K^b^* transgenic animals developed focal areas of demyelination similar to non-transgenic animals (Figure 2A and B). Few of the *D^b^* transgenic mice demyelinated (Table 1), demonstrating resistance compared to the littermate controls (Figure 2C and D) which suggests that transfer of the genomic fragments faithfully reproduced the disease susceptibility phenotypes of interest.

**Table 1:** Spinal cord pathology.

Transgene/Founder	Demyelination			
	Pos	Neg	Total	%
**Normal Littermates**	41	6	47	87
**H-2K** b	10	0	11	100^a^
**NLM/H-2K** b	51	6	67	88
**H-2D** b				
Founder 1	1	2	3	
Founder 2	1	2	3	
Founder 3	3	9	12	
Total	5	13	18	28
**H-2K** b **/D** b **α1α2**				
Founder 1	6	1	7	
Founder 2	8	1	9	
Founder 3	8	3	11	
Founder 4	4	2	6	
Founder 5	3	2	5	
Founder 6	4	2	6	
Founder 7	4	2	6	
Founder 8	2	4	6	
Total	39	17	56	70^b^ ^c^

Overall Chi Square analysis (P<0.001).

a Fisher Exact Test NLM VS H-2K^b^ (P = 0.577).

b Fisher Exact Test H-2D^b^ vs K^b^/D^b^α1α2 (P = 0.0198).

c Fisher Exact Test NLM/H-2K^b^ vs K^b^/D^b^α1α2 (P = 0.0105).

30% TMEV resistance attributed to peptide presentation.

70% TMEV resistance attributed to differences between K and D loci.

To evaluate whether factors other than peptide binding by MHC class I molecules determine disease susceptibility, we sought to remove the peptide binding and TCR interaction encoding domain as a variable in our analysis. Because the inability to present peptides to T cells would mask the hypothesized properties of interest, we chose to substitute the *D^b^* encoded antigen presenting domain α1α2 for its homologous counterpart in the *K^b^* gene. This region is known to determine the peptide binding properties of MHC I antigen presenting molecules, as well as, the specificity of MHC ligand interactions with T cell receptors [3], [20]. The resulting chimeric genomic transgene (K^b^α1α2D^b^) contained the coding sequences of the α1α2 peptide binding domain and non-coding intronic sequences from the 3′ end of intron 1, all of intron 2, and the 5′ region of intron 3 from D^b^ (Figure 1A and B). The remaining regulatory, untranslated regions and structural features of this transgene were derived from a *K^b^* genomic clone [21]. Equivalent expression of the D^b^ and the *K^b^α1α2D^b^* transgenes was observed in transiently transfected 293T cells (Figure 1C). Eight independent FVB founder lines expressing the *K^b^α1α2D^b^* transgene were established and analyzed for their susceptibility to TMEV induced demyelinating disease. Mice from 8 *K^b^α1α2D^b^* founder lines developed demyelinating lesions (Table 1), similar to transgene negative matched littermate controls (Figure 2E and F). We conclude from this analysis that the *K^b^α1α2D^b^* chimeric transgene lacks necessary determinants to provide resistance to TMEV induced demyelinating disease.

Since the level of demyelination correlates with levels of persisting virus in the CNS, we infected *D^b^* and *K^b^α1α2D^b^* transgenic mice with TMEV for 21 to 24 days to evaluate whether transgenes conferred protection from chronic virus infection. We analyzed TMEV specific transcripts from chronically infected brain and spinal cord and found that the virus persisted in both tested sublines of K^b^α1α2D^b^ mice at levels similar to levels seen in the non-transgenic FVB hosts (Figure 2G and H). In contrast, levels of virus were detected at low levels in the two evaluated D^b^ transgenic founders, one of the original 3 *D^b^* founder lines, and a fourth *D^b^* founder line expressing a *D^b^* transgene with introduced LoxP sites. The critical role of *D^b^* in viral clearance is illustrated by the deletion of *D^b^* in a version of the transgene containing LoxP sites flanking the exon encoding the transmembrane region by introduction of Cre recombinase into the mice under control of the E2a promoter. Significantly higher levels of viral transcript were observed in FVB D^b-LoxP^ Cre mice than in the FVB D^b- LoxP^ subline (Figure 2G).

### Acute and chronic CTL responses to immunodominant VP2_121–130_ are generated equivalently in H-2K^b^ α1α2D^b^ and H-2D^b^ transgenic mice

**Figure 3:**
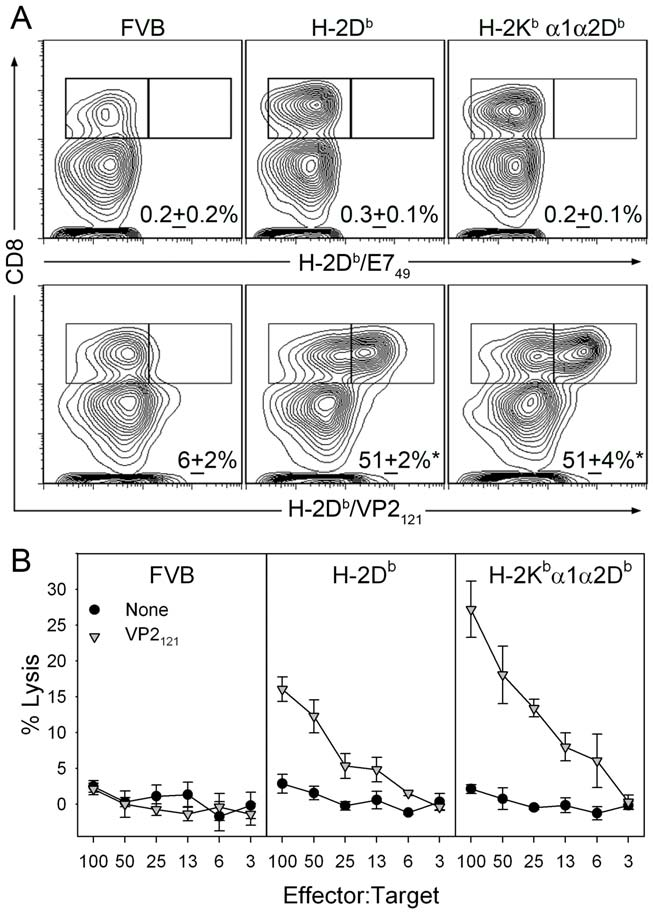
FVB K^b^α1α2D^b^ transgenic mice generate functional CTL responses against the immunodominant VP2_121–130_ peptide. (A) Control H-2D^b^/E7_49–57_ and TMEV specific H-2D^b^/VP2_121–130_ tetramer and CD8 staining of acute CD45+ lymphocytes isolated from the brains of transgenic and non-transgenic mice infected with TMEV for 6 days (* p<0.05 by ANOVA). (B) VP2_121–130_ specific killing of peptide pulsed targets by acute brain infiltrating lymphocytes isolated from H-2D^b^ and H-2K^b^α1α2D^b^ transgenic mice compared to killing of non-pulsed EL-4 cells.

As shown previously [12], the clearance of TMEV is dependent on the generation of H-2D^b^ restricted CD8 T-cell responses to the immunodominant peptide VP2_121–130_ (CD8^VP2+^). This implies that the T-cell repertoire is available to respond to this antigen and that the antigen specific CTL are able to efficiently target virus infected cells disrupting the infection. Since *K^b^α1α2D^b^* transgenic mice fail to clear virus from the CNS, we analyzed brain infiltrating lymphocytes from 6 day TMEV infected *D^b^* and *K^b^α1α2D^b^* transgenic mice for the presence of CD8+ VP2_121–130_-specific T cells. As expected, the non-transgenic FVB (H-2^q^) infiltrates were negative for D^b^-restricted VP2_121–130_-specific CD8 T cells (Figure 3A); in contrast, the *D^b^* transgenic mice developed a robust CD8+ VP2_121–130_-specific response. Surprisingly, the susceptible *K^b^α1α2D^b^* transgenic hosts developed a CD8+ response, equivalent to the response seen in the resistant D^b^ transgenic hosts, demonstrating that the VP2_121–130_ responsive CD8 T-cells were available, activated, and recruited to the brain equivalently by both resistant and the susceptible transgenic animals.

We wondered whether the presence of CD8+ T-cells specific for VP2 in the CNS suggested a defect in the ability of the T cells to target virus infected cells. We analyzed brain infiltrating leukocytes for their ability to kill VP2_121–130_ pulsed target cells. T cells from animals capable and incapable of eliminating persisting virus killed the peptide pulsed targets in vitro (Figure 3B). This indicates that the population of T cells required for viral clearance is present within the repertoire of *K^b^α1α2D^b^* transgenic mice. They are activated by virus infection, capable of killing cells presenting viral antigens, and are recruited to the infected CNS. Therefore, it appears that targeting the infected cells in vivo is deficient in *K^b^α1α2D^b^* transgenic hosts.

### Elements outside of the α1α2 coding region determine the expression levels of the MHC class I transgenes

**Figure 4:**
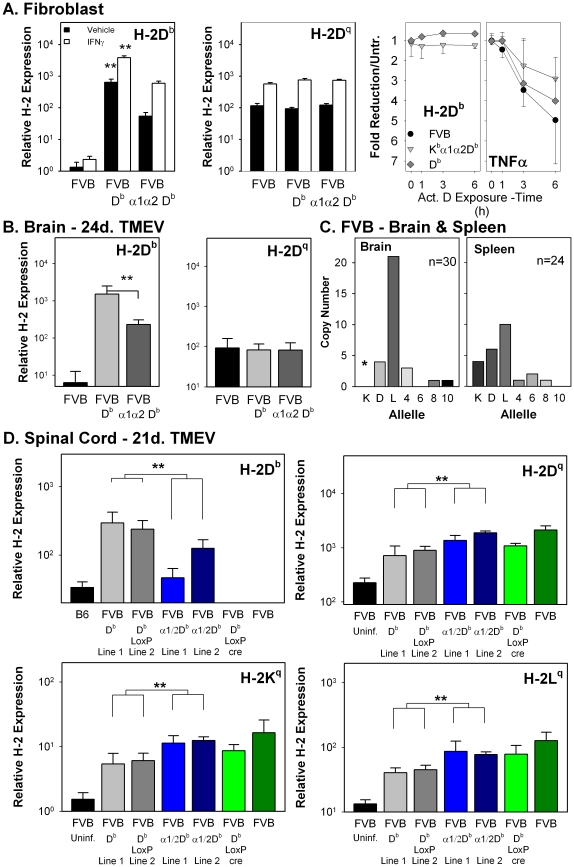
Non-equivalent mRNA expression of MHC class I genes due to elements outside of the α1α2 coding region of the transgenic D locus. (A) FVB skin fibroblast mRNA levels for H-2D^b^ and H-2D^q^ with or without treatment with IFNγ (**p<0.01 comparing D^b^ versus K^b^α1α2D^b^ without or with IFNγ). (Third panel) Transgenic mRNA stability as determined by degradation of mRNA transcripts after actinomycin D inhibition of transcription. qRT-PCR amplification was used to detect H-2D^b^ and TNFα specific transcripts from FVB D^b^ and FVB K^b^α1α2D^b^ transgenic fibroblasts previously treated with IFNγ. (B) H-2D^q^ mRNA expression in TMEV infected brain tissue from H-2D^b^ and FVB K^b^ α1α2D^b^ transgenic mice (** p<0.05). (C) Competitive RT-PCR and sequence identity for MHC specific transcripts from the brain and spleen of TMEV infected mice (* p = 0.091, Fisher Exact Test comparing the ratio of K^q^ and L^q^ sequences recovered). (D) mRNA expression of transgenic and endogenous H-2 genes (** p<0.01 by Two-way ANOVA).

We next examined the expression of the MHC class I transgenes for evidence of differential expression. Populations of skin fibroblasts were established from animals of each parental and transgenic FVB mice. The FVB K^b^α1α2D^b^ fibroblasts had significantly reduced accumulations in RNA from their class I transgene in comparison to the FVB D^b^ fibroblasts, before and after treatment with IFNγ (Figure 4A). Direct comparison was possible because the assessed transcripts shared identical sequences that were probed using quantitative PCR. In contrast, fibroblasts from FVB, FVB-D^b^, and FVB-K^b^α1α2D^b^ mice were found to have equivalent amounts of RNA from the endogenous D^q^ locus, before and after IFNγ treatment. The response of the transgenes to IFNγ appears to be equivalent to the response of the endogenous D locus based on fold-increase induced by the cytokine. The fold increase of the D^q^ (8.2±0.9) and D^b^ (11.2±2.0) genes in the FVB D^b^ fibroblast were not statistically different, similar to that observed in the FVB K^b^α1α2D^b^ fibroblasts (D^q^, 6.1±0.5 and D^b^, 6.1±0.8). We considered the possibility that the observed differences reflect variation in mRNA stability, but found no differences in mRNA stability between H-2D^b^ transcripts from the two transgenes (Figure 4A). We compared this to the less stable transcript derived from tumor necrosis factor alpha (TNFα) [22] which demonstrated a 3 to 5 fold reduction in transcript level after 6 hours of treatment with actinomycin D. We conclude that the primary functional difference observed can be attributed to the base-line expression levels of the D^b^ and K^b^α1α2D^b^ transgenes and not to their ability to respond to IFNγ.

A similar difference in expression level was observed in the brains of TMEV infected mice (Figure 4B). Again, brain tissue from FVB K^b^α1α2D^b^ transgenic mice expressed equivalent levels of endogenous D^q^ but had reduced levels of K^b^α1α2D^b^ expression compared to FVB D^b^ after TMEV infection. Expression of the endogenous K locus in brain cells from wild-type FVB mice infected with TMEV was also reduced relative to the D-region class I genes, most pronounced for the L locus (Figure 4C). In contrast, the levels of K, D, and L expression were more comparable in the spleen of infected mice. The differential pattern of expression was seen in the spinal cord of TMEV infected transgenic and wild type animals (Figure 4D). D^b^ expression was higher in two independent transgenic lines as compared to two independent K^b^α1α2D^b^ transgenic lines. Comparison of endogenous gene expression 21 days after virus infection is complicated by viral clearance in the animals expressing the D^b^ transgene relative to the other FVB mouse lines analyzed (Figure 2G). Clearance of the virus is associated with down regulation of D^q^, L^q^, and K^q^ transcripts in the D^b^ mice relative to the expression levels seen in FVB, K^b^α1α2D^b^, and D^bLoxP-cre^ mice. This expression pattern relative to virus levels is opposite to the pattern for D^b^ and K^b^α1α2D^b^ transgenes (top left panel, Figure 4D), suggesting that the functional difference in expression of the transgenes may be greater than measured in the mice 21 days after infection when virus levels are dropping in the D^b^ transgenic mice. Also noted in these experiments was the lower level of H-2K^q^ transcripts relative to D^q^ and L^q^ detected in all the mice analyzed, a finding consistent with our cDNA analysis in Figure 4C.

**Figure 5:**
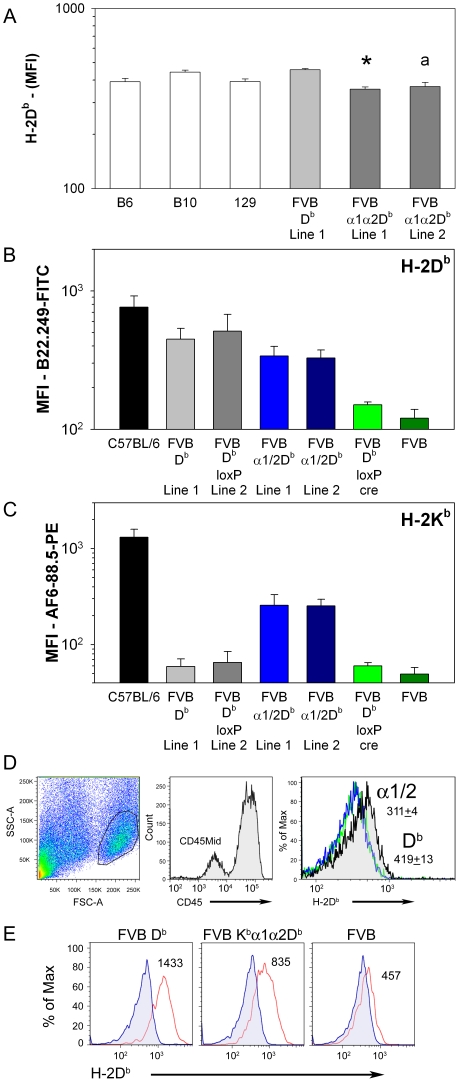
Surface expression of H-2 transgenes is regulated by genomic elements outside of sequences encoding peptide binding domains. (A) The mean fluorescence intensity (MFI) for peripheral blood lymphocytes stained with the H-2D^b^ specific antibody B22.249 from H-2^b^ haplotype mice (white) and transgenic mice (gray); (* Line 1 v. B10 or FVB D^b^, p<0.05, ^a^ Line 2 v. B10 or FVB D^b^, p<0.05). (B) Splenocytes from mice infected with TMEV for 21 days were isolated and tested by flow cytometry for H-2D^b^ expression levels using B22.249.R1 labeled with FITC. Data are expressed as the MFI of the FITC labeled cells (* p<0.05, Two-way ANOVA). (C) The same splenocytes in (B) were co-stained with AF6-88.5 labeled phycoerythrin to determined expression of the H-2K^b^ present on the chimeric K^b^α1α2D^b^ molecule. Data are expressed as MFI of PE labeled cells (* p<0.001, Two-way ANOVA). (D) Brain resident cells from FVB, FVB D^b^ and FVB K^b^α1α2D^b^ mice were isolated from naïve mice and analyzed by flow cytometry. Side-scatter and forward-scatter were analyzed for the presence of mononuclear resident cells using a lymphocyte gate. Resident antigen-presenting cells were further analyzed by a CD45 mid-level staining gate and assessed for expression of MHC class I. FVB (green), FVB D^b^ (black) and FVB K^b^α1α2D^b^ (blue) brain cells were assessed for H-2D^b^ expression by FACS (* p<0.001, t-test). (E) Brain cells isolated from 6 day TMEV infected mice were gated as in (D) and assessed for changes in H-2D^b^ expression by FACS. Data are the MFI for H-2D^b^-FITC.

Next, we assessed the expression of the transgene and endogenously encoded class I genes at the protein level. The mean fluorescence intensity of class I molecules expressing the B22.249 (D^b^) defined epitope shared by D^b^ and K^b^α1α2D^b^ was comparable in peripheral blood mononuclear cells for all the tested mouse lines, although a slight, but statistically significant lower expression was seen for the chimeric transgene encoded molecules (Figure 5A). A similar expression pattern was seen using spleen cells when comparing independent founder lines (Figure 5B). Expression of the K^b^α1α2D^b^ transgene was confirmed in these mice by using an antibody [23] with a reactivity pattern dependent, in part, on the α3 region of K^b^ (Figure 5C).

The central element of our hypothesis is that the expression of class I loci in tissues within the body are not equivalent and that cells, which do not adequately express class I antigen presenting molecules, can provide safe harbor for infecting virus despite the presence of infiltrating, activated, anti-viral T cells. In the specific case of CNS infection by TMEV, we hypothesize that the K locus is not universally expressed by cells infected by TMEV, while the D locus is. Therefore, we next sought to identify cells which under express K^b^α1α2D^b^ relative to D^b^ during virus infection. One limitation with this approach is that there are many different cell types in the CNS, and our hypothesis only specifies that some cells need to under express H-2K. Therefore, this hypothesis will be difficult to assess systematically. Nevertheless, using a candidate approach, we examined class I expression by microglial cells, a brain resident cell population that can be recovered from normal and infected brain for analysis by flow cytometry [24]. Microglial cells can be discriminated from infiltrating leukocytes and other resident brain cells by their intermediate expression of the leukocyte marker CD45. We enriched CD45 positive cells from brain homogenates using percol gradients and analyzed the CD45 intermediate cells for expression of D^b^ and K^b^α1α2D^b^ before (Figure 5D) and after (Figure 5E) TMEV infection. H-2D^b^ is expressed at a significantly higher level on microglial cells than is K^b^α1α2D^b^. Following infection with TMEV, expression of D^b^ is uniformally increased, while the increase of expression of K^b^α1α2D^b^ is more variable. Importantly, a portion of the microglial cells isolated from TMEV infected K^b^α1α2D^b^ transgenic mice do not express levels of the encoded H-2 antigen presenting molecules beyond the median level of the negative control (shaded in Figure 5E). This expression pattern is consistent with our hypothesis that determinants mapping outside the peptide binding domains of the K and D class I genes differentially regulate tissue specific expression and that a population of cells in the CNS may not adequately express the relevant MHC encoded antigen presenting molecule needed to clear virus.

## Discussion

In this report we show, in agreement with previous reports [25], [26], that the ability of the *H-2D^b^* gene to incite a virus specific CTL response maps to the coding sequences of the peptide binding domain of the class I glycoprotein. However, we also found that induction of a virus specific CTL response that is recruited into the infected CNS is not sufficient to clear virus infection and protect the mice from virus induced demyelination [27]. Another, yet to be defined, attribute of the *D^b^* gene is necessary for effective immunity to persistent virus infection. This attribute of *D^b^* is not shared by the *K^b^* gene and maps outside of the peptide binding domain coding sequences and adjacent intron sequences. We demonstrated in earlier studies using bone marrow chimeras that activated brain infiltrating CTL must be able to recognize virus on infected cells in order to clear the virus infection [27]. One possibility is that the H-2K antigen presenting molecules are not expressed sufficiently on populations of TMEV infected cells in the CNS, preventing the effective clearance of the virus by K restricted CTL.

The necessary structural sequences for directing effective antiviral immunity are present within the 8 kb genomic DNA fragment introduced as a transgene in our study, as four founder lines prepared from susceptible FVB mice acquired a resistant phenotype using demyelination (Table 1) or viral clearance (Figure 2) as criteria. In contrast, the eight founder lines prepared using the K^b^α1α2D^b^ transgene displayed a susceptible phenotype. Therefore, a systematic analysis of recombinant transgenes should allow identification of the critical sequences determining the functional differences between these two classical class I genes.

Brahic and colleagues reported that a transgene encoding a similar chimeric glycoprotein comprised of the α1α2 domain of D^b^ in the context of the α3, transmembrane, and cytoplasmic regions of K^b^ was able to clear TMEV infection from the CNS [28]. The chimeric gene used in that study contained a larger segment of D^b^ non-coding sequences than did the chimeric transgene used in our study. One possibility is that regulatory sequences determining the ability of the *D* class I gene to direct effective sterilizing immunity against the virus are located within the additional *D^b^* sequences in the Brahic construct. Another possibility is that a fortuitous integration event bestowed a wider expression phenotype on the transgenic mouse used in that study. Our finding that 8 different K^b^α1α2D^b^ founder lines expressed similar demyelinating phenotypes known to be related to inability to clear chronic virus infection provides confidence in our conclusion that structural components of the transgenes, and not fortuitous integration events, determined the different phenotypes of the transgenic animals we studied. The ability of our chimeric transgene to promote the activation and recruitment of virus specific CTL to the infected CNS in a manner equivalent to the phenotype bestowed by the parental *D^b^* transgene has led us to a different conclusion from the Brahic group. We conclude that the ability of peptide binding domain to present critical viral peptide antigens and elicit cellular immunity is not sufficient to direct effective antiviral immunity to the TMEV picornavirus and that properties distinguishing *D* region class I genes from the *K* locus genes determine this difference.

We had previously noted using immunohistology that the K and D region proteins of the *H-2^q^* haplotype are differentially expressed in TMEV infected brain tissues during acute and chronic infection [29]. In those studies, we found early up regulation of H-2K and H-2D protein in the brain. While H-2D expression was maintained during the chronic phase of infection, expression of H-2K diminished. Here, we have extended our analysis of the expression of *H-2^q^* haplotype genes during TMEV infection at the RNA level. We find that *H-2L^q^* is the most prominently expressed class I gene in TMEV infected mice, with minimal expression *of H-2K^q^*. Our comparison of the chimeric and D^b^ transgenes showed that D^b^ is expressed at substantially higher levels than the chimeric gene in TMEV infected CNS of transgenic mice, a pattern that mimics the endogenous K^q^ and L^q^ genes. H-2L^q^ and H-2D^b^ are close evolutionary relatives as indicated by dendogram analyses based on their gene sequences [19]. Their notation as L and D locus alleles is an accident of mouse nomenclature. We have argued that all the classical D region genes can move into the D and L locus positions on the chromosome by unequal recombination [30], and therefore, can be considered part of the same allelic series, although structural regulatory differences derived from two ancient loci now mixing by the polymorphic gene organization may be retained in mouse populations. According to our hypothesis, D^b^ is protective because it is highly expressed in infected tissues and presents viral peptides efficiently, while L^q^, also highly expressed, does not effectively present viral peptides. *H-2D^q^* expression, while measurable, does not appear to be at the level of H-2L^q^. How other *D* region genes in the mouse are expressed, including those of the resistant H-2^d^ and H-2^k^ haplotypes, remains to be determined.

An important insight emanating from our studies is that the classical MHC I genes do not appear to function equivalently throughout the body. While these molecules are all capable of binding endogenously generated peptides and presenting both foreign and self antigens to CD8+ T cells, at least some of the *K* locus alleles (*K^q^* and *K^b^*) are expressed at lower levels than their D region counterparts (*L^q^*, *D^b^*, and even *D^q^*) by fibroblasts and some cells in the CNS. All these MHC I genes appear to be expressed more similarly in some tissues outside of the CNS, most notably spleen cells and by brain infiltrating leukocytes during infection. The implication is that regulation of MHC I gene expression in the CNS and in spleen cells differs in ways that requires more than the up regulation of a single gene regulatory factor not normally expressed in CNS tissues. Our model provides a way to identify the elements governing the regulatory mechanisms active in the CNS.

Although we do not know the precise mechanism responsible for differential regulation of K and D genes in the mouse, both of our transgenes are responsive to IFNγ, but differ in basal expression in various tissues. This expression pattern is consistent with previous findings demonstrating that distinct transcriptional pathways regulate basal and activated MHC class I expression [31] and locus specific differences in the structure of MHC class I promoters [25], [26] reflected in patterns of transcription factor binding [32], [33]. The precise details of the mechanisms that determine differential expression of class I genes in different species may not be the same.

Finally, our finding that MHC I loci have different inherent abilities to direct effective class I mediated cellular immunity to viruses provides a model for understanding the strong selection differences implicit in the structures of polymorphism of class I genes in human and chimpanzee populations. Whereas the *B* locus alleles are vibrantly selected for diversity in the coding blocks for their peptide binding sites, *A* and C locus alleles are less so. The *B* locus in humans and the *D* loci in mice occupy orthologous positions within their respective MHC [34] raising the possibility that these genes may share canonical regulatory motifs and expression patterns. Whether locus differences in polymorphism can be traced to differential regulation of the primate A, B, and C loci in the CNS or elsewhere in the body remains to be determined. Nonetheless, the implication is that the *A*, *B*, and *C* loci are not functioning equivalently throughout the body. This raises provocative questions about the wisdom of selecting the less polymorphic *HLA-A* locus antigen presenting proteins as targets for vaccine development. The requirements for initiating an immune response and for resolving infection are different. The absence of expression of *A* locus proteins in key tissues (e.g. sites of infections) could promote conditions of chronic inflammation without effective resolution of immunity at targeted tissues. We propose that differences in gene regulation may be the underlying reason why *A* and *C* diversity is lagging behind *B* diversity in human populations.

## Materials and Methods

### Ethics statement

This study was carried out in strict accordance with the recommendations in the Guide for the Care and Use of Laboratory Animals of the National Institutes of Health. The protocols were approved by the Institutional Animal Care and Use Committee of Mayo Clinic (#A25704 and #A12304). All mice were anesthetized with isoflurane prior to intracranial virus infection.

### Mice

C57BL/6 mice were obtained from Jackson Laboratories (Bar Harbor, ME, USA). B10, 129 and FVB mice were obtained from the Mayo Clinic Transgenic Core Facility.

### Virus infection

Virus infection was introduced into the CNS via intracerebral inoculation with 2×10^5^ PFU of the Daniel's strain of Theiler's murine encephalomyelitis virus (TMEV). Acute infection with TMEV was analyzed at day 6 and all chronically infected animals at greater than 45 days. All animals were housed and cared for according to institutional and NIH guidelines for animal care and use.

### Transgenic mice

The generation of FVB K^b^ and FVB D^b^ transgenic mice was described previously [12]. FVB K^b^α1α2D^b^ transgenic mice were generated by the Mayo Transgenic Core facility. To generate the chimeric construct a Sal I site was introduced into a Hind III-EcoRI fragment of H-2K^b^ at genomic position 136. A Sal I/XbaI PCR fragment was generated from a cloned H-2D^b^ vector [35] and engineered into the H-2K^b^ backbone vector.

Function was verified in 293T cells by transfection and FACS. Briefly, 293T cells were plated in 6 well plates one day prior to transient transfection with GFP and the H-2K^b^, H-2D^b^ and H-2K^b^α1α2D^b^ constructs using Fugene 6 transfection reagent (Roche Diagnostics Corporation, Indianapolis, IN). Twenty-four hours later cells were trypsinized and stained with excess H-2D^b^ specific antibody (B22.249.R1). A secondary anti-mouse IgG phycoerythrin antibody (Accurate Chemical, Westbury, NY) was used was used at 10 µg/mL to detect H-2D^b^ expression. MHC I transgenic constructs were injected into FVB/Cr blastocysts to generate H-2K^b^, H-2D^b^ and H-2K^b^α1α2D^b^ transgenic mice.

We (AJJ) generated the FVB/N-D^b^ LoxP mouse to achieve cell-specific deletion of the H-2D^b^ class I molecule through modification of our H-2D^b^ transgene [12]. Using conventional molecular biology techniques, we inserted LoxP sites that flank the transmembrane exon of the D^b^ class I gene (exon 5). This drives D^b^ class I gene expression in the FVB/N strain (H-2^q^). Transgenic FVB/N-D^b^ LoxP mice elicited normal CNS infiltrating D^b^:VP2 _121–130_ epitope specific CD8 T cell responses during acute TMEV infection (data not shown). To verify function of the LoxP transgene, we bred the FVB/N-D^b^ LoxP mice to the FVB/N-Tg (E2a-cre) C5379Lmgd/J mouse (Jackson: 003314). This line carries a Cre transgene under the control of the adenovirus E2a promoter that targets expression of Cre recombinase in a wide variety of tissues. Thymocytes isolated from progeny FVB/N-D^b^ LoxP mice that expressing the Cre transgene under the E2a promoter had deactivation of D^b^ class I expression (data not shown).

### Histology

Chronically infected mice were perfused and fixed with Trump's fixative before dissociated spinal cord sections were fixed with osmium tetroxide, dehydrated, embedded in glycol methacrylate and sectioned as described previously [36]. Spinal cord sections were analyzed for the presence of demyelination characterized by the presence of focal lesions composed of infiltrating lymphocytes, myelin debris and de-nuded axons.

### Semi-Quantitative RT-PCR for TMEV, cytokine and MHC class I mRNA

The 7900HT Fast Real-Time PCR System (Applied Biosystems, Carlsbad, CA, USA) was used to quantify viral RNA infected brain and spinal cord homogenates from mice inoculated with TMEV-wt or TMEV-L/OVA. RNA was isolated using TRIzol Reagent (Invitrogen, Carlsbad, CA, USA) and reverse transcribed using the Superscript cDNA synthesis kit (Invitrogen). Reaction was set up using the Fast SyBR Green Master Mix Kit (Applied Biosystems). cDNA was amplified using primers specific for mouse actin (F – 5′CTGGCACCACACCTTCTACAATGAGCTG and R– 5′GCACAGCTTCTCTTTGATGTCACGCACGATTTC) and for viral protein 2 (VP2) of TMEV (F-5′TGGTCGACTCTGTGGTTACG and R-5′ GCCGGTCTTGCAAAGATAGT). Cycling conditions were as follows: 50°C for 2 minutes, 95°C for 10 minutes followed by 40 cycles of 95°C at 15 seconds then 55°C for 1 minute. Amplification curves and crossing point thresholds were based on SYBR Green incorporation. Samples were normalized to actin and data are reported as fold increase over background or the appropriate control strain.

Primers specific for H-2D^b^ (5′GAAACACAGAAAGCCAAGGGCCAA and 5′AGTCCGACCCCAAGTCACAGCCAG), H-2D^q^ (5′GATCACGCAGATCGCCAAGGACAAT and 5′CGTGCAACCCCACGTCACAGCCGTACATCC), H-2L^q^ (5′GTCCCGCAGGCACTCACACGATCCAG and 5′CCGTCGTATGCGTACTGCTCGTACCC) and H-2K^q^ (5′ACGACACTGAGTTGGTGCGCTTCGAC and 5′ACTCTGCTCATTGTCCTTGGCGATCT) were used to evaluate H-2 expression in fibroblast, spinal cord and brain by semi-quantitative real-time RT-PCR. Samples were normalized to actin and fold change was calculated relative to non-transgenic or to background amplification of mice not having the amplified allele.

To determine the relative contribution of different H-2 alleles to the overall expression of MHC class I in the CNS, we used a competitive PCR technology to amplify multiple MHC class I alleles and then verified their identity by sequencing. We used flanking primers that were conserved across several alleles but spanned areas of diversity that allowed us to discriminate individual alleles. We added flanking BamHI sites to each primer so that concatamers could be generated from amplified fragments (Forward 5′CATATAATAATGGATCCTACTACAACCAGAGC, Reverse 5′GTATATTATCGGATCCGTACCCGCGGAGGAG). Concatamers were cloned and sequenced using standard techniques.

### Skin fibroblasts

Skin fibroblast lines were derived from ear punches obtained from FVB, FVB D^b^ and FVB K^b^α1α2D^b^ transgenic mice according to previously described methods [37].

### Analysis of mRNA stability

mRNA stability was assessed using previously described techniques [38]. Skin fibroblasts derived from FVB and transgenic FVB D^b^ and FVB K^b^α1α2D^b^ mice were treated with 1 µg/mL of recombinant mouse interferon gamma (R&D Systems, Minneapolis, MN) in triplicate. Twenty-four hours later cells were either left untreated or treated with 2.5 mg/mL of actinomycin D (Sigma-Aldrich, St. Louis, MO) for 1, 3 and 6 hours. After actinomycin D exposure RNA was extracted using TRIzol reagent. Real-time RT-PCR assessment of fold reduction in expression of H-2D^b^ and TNFα (Forward primer 5′GGATGAGAAGTTCCCAAATGGCCTC and Reverse primer 5′ GCTCCTCCACTTGGTGGTTTGCTA) was performed using actin as a normalization control. Data were expressed as the fold reduction in expression from the untreated control sample.

### Analysis of central nervous system derived lymphocytes

Brain and spinal cord infiltrating lymphocytes from TMEV infected mice were recovered using previously described techniques and were analyzed by flow cytometry [10], [39]. Anti-CD45 (30-F11) and anti-CD8 (53-6.7) antibodies were obtained from Ebiosciences (San Diego, CA, USA). FITC labeled anti H-2D^b^ (B22-249R1; Accurate, Westbury, NY, USA) and PE-labeled anti H-2K^b^ (AF6-88.5; BD Pharmingen, San Jose, CA, USA) were used to assess MHC class I expression on PBMC, splenocytes and brain infiltrating cells. All antibodies were used in excess at concentration of 10 µg/mL. VP2_121–130_/H-2D^b^ tetramers were kindly provided by the NIH Tetramer Core Facility at Emory University (Atlanta, GA, USA) and were used at a concentration of 12 µg/mL. HPV16 E7_49–57_/H-2D^b^ tetramers (Becton-Dickinson, San Jose, CA) were used as a non-specific control according to the manufacturers guidelines. Samples were analyzed on a BD LSR II flow cytometer (BD Biosciences, San Jose, CA) and analyzed using FloJo software (Ashland, OR). Single color stained splenocytes were used as compensation controls. CD45-allophycocyanin staining was used to discriminate brain derived microglia that moderately autofluoresce in the FL1 channel from other brain derived cells. Comparisons between the three transgenic strains were performed on the same population of CD45 gated cells. Cytotoxic lymphocyte killing of VP2_121_ peptide pulsed targets was assessed by chromium release assay using EL4 cells pulsed with 1 µg/mL of peptide [10].

### Statistics

Normally distributed data were analyzed by ANOVA or t-test. Data failing the normality test were analyzed by ANOVA on ranks or Rank-sum test. Pairwise comparisons for ANOVA were performed using the Holm-Sidak method. Significance was determined by p less than 0.05.
